# Supplementary material for: Bedrock radioactivity influences the rate and spectrum of mutation
Source: eLife. 2020 Nov 30;9:e56830. doi: 10.7554/eLife.56830 (PMC7723406; doi:10.7554/eLife.56830)
Supplement: Transparent reporting form [file elife-56830-transrepform.pdf]

eLIFE
1st Floor
24 Hills Road
Cambridge CB2 1JP, UK
P 01223 855340
W elifesciences.org
T @elife

eLife's transparent reporting form

We encourage authors to provide detailed information *within their submission* to facilitate the interpretation and replication of experiments. Authors can upload supporting documentation to indicate the use of appropriate reporting guidelines for health-related research (see EQUATOR Network), life science research (see the BioSharing Information Resource), or the ARRIVE guidelines for reporting work involving animal research. Where applicable, authors should refer to any relevant reporting standards documents in this form.

If you have any questions, please consult our Journal Policies and/or contact us: editorial@elifesciences.org.

Sample-size estimation

- You should state whether an appropriate sample size was computed when the study was being designed
- You should state the statistical method of sample size computation and any required assumptions
- If no explicit power analysis was used, you should describe how you decided what sample (replicate) size (number) to use

Please outline where this information can be found within the submission (e.g., sections or figure legends), or explain why this information doesn't apply to your submission:

We prospected 58 sites with different level of radioactivity to find subterranean isopods species. With the information collected, we selected pairs of closely related species, each pair comprised of one species exposed to a low level of natural radioactivity and the other one exposed to a high level natural radioactivity (3X increase). We identified a maximum of 7 such pairs and selected all of them. We sequenced the transcriptome of 8 individuals per species to characterize each population mutational spectrum. A design with 7 pairs of species that form 7 independent *in naturae* replicates to study the impact of bedrock radioactivity is expected to be statistically powerful and unprecedented.

Replicates

- You should report how often each experiment was performed
- You should include a definition of biological versus technical replication
- The data obtained should be provided and sufficient information should be provided to indicate the number of independent biological and/or technical replicates
- If you encountered any outliers, you should describe how these were handled
- Criteria for exclusion/inclusion of data should be clearly stated
- High-throughput sequence data should be uploaded before submission, with a private link for reviewers provided (these are available from both GEO and ArrayExpress)

Please outline where this information can be found within the submission (e.g., sections or figure legends), or explain why this information doesn't apply to your submission:

All these informations can be found in the Methods section of the article. The number of specimens collected and sequenced are described in the subsection "sampling" (l.184). The rationale for the exclusion of 2 samples is explained in the subsection "Received dose" (l.235). All sequences have been deposited on the ENA, and alignments on Zenodo, accession numbers and links are indicated in the section "Data availability" (l. 368).

Statistical reporting

- Statistical analysis methods should be described and justified
- Raw data should be presented in figures whenever informative to do so (typically when N per group is less than 10)
- For each experiment, you should identify the statistical tests used, exact values of N, definitions of center, methods of multiple test correction, and dispersion and precision measures (e.g., mean, median, SD, SEM, confidence intervals; and, for the major substantive results, a measure of effect size (e.g., Pearson's r, Cohen's d)
- Report exact p-values wherever possible alongside the summary statistics and 95% confidence intervals. These should be reported for all key questions and not only when the p-value is less than 0.05.

Please outline where this information can be found within the submission (e.g., sections or figure legends), or explain why this information doesn't apply to your submission:

Statistical methods are described in the subsection "Statistical analyses" (l.355). N, p.values and other statistics are indicated in the tables 1 (p 7) and 2 (p.9) of the "Results" part. For tests between substitution rates and radioactivity, 95% confidence intervals of the pGLS models are shown in the Figure 2 (p.6).

(For large datasets, or papers with a very large number of statistical tests, you may upload a single table file with tests, Ns, etc., with reference to sections in the manuscript.)

Group allocation

- Indicate how samples were allocated into experimental groups (in the case of clinical studies, please specify allocation to treatment method); if randomization was used, please also state if restricted randomization was applied
- Indicate if masking was used during group allocation, data collection and/or data analysis

Please outline where this information can be found within the submission (e.g., sections or figure legends), or explain why this information doesn't apply to your submission:

eLife Sciences Publications, Ltd is a limited liability non-profit non-stock corporation incorporated in the State of Delaware, USA, with company number 5030732, and is registered in the UK with company number FC030576 and branch number BR015634 at the address 1st Floor, 24 Hills Road, Cambridge CB2 1JP | August 2014

eLIFE
1st Floor
24 Hills Road
Cambridge CB2 1JP, UK
P 01223 855340
W elifesciences.org
T @elife

We selected pairs of sister species to have a 3X increase of radioactivity between the two partners of the pair. Other than that, we did not have to allocate samples into groups.

Additional data files ("source data")

- We encourage you to upload relevant additional data files, such as numerical data that are represented as a graph in a figure, or as a summary table
- Where provided, these should be in the most useful format, and they can be uploaded as "Source data" files linked to a main figure or table
- Include model definition files including the full list of parameters used
- Include code used for data analysis (e.g., R, MatLab)
- Avoid stating that data files are "available upon request"

Please indicate the figures or tables for which source data files have been provided:

Source data have been deposited on Zenodo (https://doi.org/10.5281/zenodo.3356835):

- Figure 1 : The 14 samples were chosen based on a large prospection of 58 sites. Coordinates, alpha radioactivity measurement, species found in these 58 sites and accession numbers for 16S sequences of collected specimens are described in the Table 1 of the Zenodo document.

- Figure 2, Table 1 and Table 2 : The Received Dose of radioactivity have been computed based on the measurement of all radionuclides in sediment. The raw measurement of these radionuclides are indicated in the Table 2 of the Zenodo document. The computational method to estimate radionuclide activities are described in the Table 3, and the resulting estimated radionuclide activities are indicated in Table 4.

- Figure 3 and Table 2 : The counts for each mutation type are indicated in the Table 6 of the Zenodo document and the resulting relative proportions of each mutation type are indicated in the Table 8. The counts and relative proportions computed on third positions only are available in the tables 7 and 9 respectively.

eLife Sciences Publications, Ltd is a limited liability non-profit non-stock corporation incorporated in the State of Delaware, USA, with company number 5030732, and is registered in the UK with company number FC030576 and branch number BR015634 at the address 1st Floor, 24 Hills Road, Cambridge CB2 1JP | August 2014
